# A unique increase in prefrontal gray matter volume in hoarding disorder compared to obsessive-compulsive disorder

**DOI:** 10.1371/journal.pone.0200814

**Published:** 2018-07-16

**Authors:** Satoshi Yamada, Tomohiro Nakao, Keisuke Ikari, Masumi Kuwano, Keitaro Murayama, Hirofumi Tomiyama, Suguru Hasuzawa, Osamu Togao, Akio Hiwatashi, Shigenobu Kanba

**Affiliations:** 1 Department of Neuropsychiatry, Graduate School of Medical Sciences, Kyushu University, Fukuoka, Japan; 2 Department of Clinical Radiology, Graduate School of Medical Sciences, Kyushu University, Fukuoka, Japan; Chiba Daigaku, JAPAN

## Abstract

**Background:**

Hoarding disorder (HD) is a disease concept newly presented in DSM-5. As far as we know, no studies have examined the structural changes relevant to hoarding by applying the diagnostic criteria of HD in DSM-5. In the present study, we aimed to find abnormalities in gray matter (GM) structures of patients with HD.

**Methods:**

Seventeen patients who met the DSM-5 criteria for HD, 17 obsessive-compulsive disorder (OCD) patients, and 17 healthy controls (HCs) participated in this study. All participants underwent MRI scanning of the brain by a 3.0-Tesla MRI scanner. In a voxel-based morphometric procedure, preprocessed GM structural images were used to compare the three groups. Thereafter we investigated the correlation between the clinical data (age of onset, symptomatic severity) and GM volume.

**Results:**

The HD group showed a significantly increased GM volume compared to the OCD and healthy control groups (p<0.05) in both Brodmann area (BA)10 and BA11. There was no significant difference between OCD and healthy control groups. No significant correlation between the clinical data including age of onset, symptom severity score, and GM volume was observed in HD and OCD groups.

**Conclusions:**

The results might help to explain the inconsistency of previous studies. As with OCD, HD is considered to have cognitive dysfunction as its basis. This result is convincing after considering the clinical features of HD and suggested that structural abnormalities in the prefrontal regions might relate to the pathophysiology of HD.

## Introduction

Hoarding disorder (HD) is a disease concept newly presented in DSM-5. Patients with HD find it difficult to throw away possessions, regardless of the actual value, and organize those things. As a result, the possessions overflow the living space, and hinder living functions. Extreme forms of this behavior can fill a living space and cause sanitation problems. Moreover, hoarding symptoms might cause injuries due to fires and collapse, which can have a great impact not only on the person themselves and their family but also on neighboring residents.

To date, the neurobiological mechanisms of HD haven't been investigated in detail. A study of human brain lesions was suggested that damage in the prefrontal region was associated with collecting behavior[1]. Furthermore, several studies have reported a correlation between hoarding symptoms and specific brain regions using neuroimaging technology. Functional neuroimaging methods, a positron emission tomography research study, revealed that obsessive-compulsive disorder (OCD) patients with compulsive hoarding had significantly lower glucose metabolism in the posterior cingulate gyrus and cuneus than normal control subjects[2]. A functional magnetic resonance imaging (fMRI) study revealed that OCD patients who were asked to imagine discarding various items demonstrated significantly greater activation than controls in left precentral gyrus and right orbitofrontal cortex[3]. Another fMRI study reported that the OCD patients with prominent hoarding symptoms showed greater activation in the bilateral anterior ventromedial prefrontal cortex (VMPFC) than patients without hoarding symptoms and healthy controls in response to a hoarding-related anxiety provocation[4]. During a hoarding-relevant decision-making task, patients with hoarding symptoms who met the clinical criteria of HD outlined by Frost and Hartl[5] and proposed for DSM-5[6] exhibited a biphasic abnormality in the insula and anterior cingulate cortex (ACC) [7]. Furthermore, several structural studies using voxel-based morphometry (VBM) indicated the association between hoarding symptom scores in OCD and gray matter (GM) volume in the left caudate[8], the left Brodmann area(BA)6[9], the left lateral orbitofrontal cortex (OFC) and the right parahippocampal gyrus[10]. These findings, however, are inconsistent. In addition, most of the previous studies focused on compulsive hoarding as a subtype of OCD. Only one fMRI study reveals that HD patients, diagnosed by DSM-5 criteria, exhibited significantly greater activity than controls in the ACC and right dorsolateral prefrontal cortex during the conflict monitoring and response inhibition conditions in the Go/No-Go task[11]. There are, as far as we know, no studies that have examined the structural changes relevant to hoarding by applying the diagnostic criteria of HD in DSM-5.

In the present study, we aimed to find abnormalities in GM structures of patients with HD by using the criteria of HD in DSM-5. To our knowledge, this is the first VBM study targeting HD patients diagnosed with DSM-5 criteria. Although previous findings are inconsistent, we hypothesized that HD patients exhibit peculiar changes in GM volume in the prefrontal region compared to OCD patients and healthy controls because of its association with collecting behavior based on a previous human regional study[1].

## Materials and methods

Seventeen patients who met DSM-5 criteria for HD, 17 OCD patients, and 17 healthy controls(HCs) participated in this study. OCD patients and HCs were matched by age and sex to HD patients. The patients with HD and OCD were recruited from outpatients and inpatients of the Department of Neuropsychiatry, Kyushu University Hospital, Japan, and the HCs were recruited from the local community. The study was approved by an ethics committee of Kyushu University and each participating patient provided written informed consent after receiving a complete description of the study, which was approved by the institutional review board.

Diagnoses of HD and OCD were confirmed using the Structured Interview for Hoarding Disorder (SIHD) [12] and the Structured Clinical Interview (SCID-I) of DSM-IV by trained psychiatrists, respectively. SIHD was obtained directly from the original authors and translated into Japanese. The HCs were without comorbid axis I diagnoses including HD and OCD and had no history of neurological illness, other disorders of the central nervous system, or history of substance abuse.

The HD patients were assessed for the severity of their hoarding symptom using the Saving Inventory-Revised (SI-R) [13] [14]and the Clutter Image Rating (CIR) [15]. The SI-R is a 23-item self-administered questionnaire requesting a response on a 0–4 scale (range 0–92), We used a Japanese version of SI-R translated by Tsuchiyagaito et al. [13]. The CIR is a visual assessment composed of a series of nine photographs that specifically assess the severity of clutter in the three main rooms (kitchen, living room, and bedroom) in a general home environment. Global symptom severity in OCD patients was assessed using the Yale-Brown Obsessive Compulsive Scale (Y-BOCS) [16]. The HD and OCD patients were also evaluated for obsessive-compulsive symptoms using the Dimensional Yale-Brown Obsessive Compulsive Scale (DY-BOCS) [17], which divides obsessive–compulsive symptoms into six distinct dimensions: aggression/checking, sexual/moral/religious, symmetry/ordering/counting, contamination/washing, hoarding and miscellaneous.　Also, the patient groups completed the Hamilton Depression Rating Scale (HDRS) and Hamilton Anxiety Rating Scale (HARS) to assess the severity of depression and anxiety. HD patients who had history of neurological illness, other disorders of the central nervous system, or history of substance abuse were excluded.

In order to exclude secondary hoarding symptoms, OCD patients who showed any hoarding symptoms (i.e., patients whose hoarding symptom scores on DY-BOCS were not less than 1 point) were excluded from the study. Also, for the purpose of eliminating OCD patients with atypical pathology such as “not just right feeling” rather than anxiety, OCD patients with atypical symptoms (such as sexual/ moral/religious, symmetry/ordering/counting) as the main symptoms were excluded. OCD patients who had comorbid axis I diagnoses and histories of neurological illness, other disorders of the central nervous system, or substance abuse were excluded.

All participants underwent MRI scanning of the brain on a 3.0-Tesla MRI scanner (Achieva TX, Philips Healthcare, Best, The Netherlands) with an 8-channel head coil at the Department of Radiology, Kyushu University. T1-weighted images were acquired with a 3D T1-weighted turbo field echo sequence with the following parameters: repetition time (TR) = 8.2 ms, echo time (TE) = 3.8 ms, flip angle = 8°, matrix = 240×240, T1 inversion time = 1026ms, field of view (FOV) = 240×240 mm, number of signal averages (NSA) = 1, slice thickness = 1 mm, number of slices = 190, and scan time = 320 s.

Acquired images were first converted from DICOM to NifTI-1 format by using the dcm2nii software at MRIcron (https://www.nitrc.org/projects/mricron). Preprocessing and quality check of data were performed with SPM12 software (Functional Imaging Laboratory, Wellcome Trust Centre for Neuroimaging, Institute of Neurology at University College London, UK; http://www.fil.ion.ucl.ac.uk/spm/) running on MATLAB R2011b (Mathworks Inc., Sherborn, MA, USA).

Prior to preprocessing, anterior commissure-posterior commissure orientation was conducted on all T1-weighted data. Each image was segmented into gray matter (GM), white matter (WM), and cerebrospinal fluid (CSF) using the segment function of SPM12. Subsequently, the segmented GM images were spatially normalized using the diffeomorphic anatomical registration through an exponentiated lie algebra (DARTEL) algorithm. DARTEL templates were generated from all the MR images of the participants. After spatial normalization, the GM images were modulated by Jacobian determinants, and smoothed with an 8 mm full width at half maximum (FWHM) Gaussian kernel. For this preprocessing, default parameters were used except for the affine regularization. Because the subjects are all Japanese, we used the East Asian template for affine regularization. Total intracranial volume was calculated by summing up gray matter volume (GMV), white matter volume (WMV), and cerebrospinal fluid volume using the “Tissue Volumes” function of SPM12.

We also calculated the regional gray matter volume in two regions of interest (ROIs). For this, we used normalized but not smoothed images because signal intensities can vary with smoothed images, resulting in inaccurate volume. ROIs were generated from the Automatic Anatomic Labeling (AAL) tool[18] [19] included in the Wake Forest University WFU PickAtlas[19]. We employed BA10 and BA11 as ROIs. We selected these areas in response to ANOVA results. We made masks of these regions using PickAtlas, and obtained the gray matter volume of each region using a Matlab script written by Ridgway (http://www.cs.ucl.ac.uk/staff/g.ridgway/vbm/get_totals.m).

After preprocessing, voxel-based analysis of variance (ANOVA) was carried out in order to investigate the presence of regional GM volume differences among the three groups (HD, OCD and HCs) using SPM12. The subject's age at the time of MRI scanning, sex and total intracranial volume (TIV) were entered as nuisance covariates.

Then, we conducted analysis of covariance (ANCOVA) to assess the regional gray matter volumes of the three groups (HD, OCD, HCs) using JMP^®^ 13 (SAS Institute Inc., Cary, NC, USA).

Using the formula *y*_*i*_ *= β*_*0*_ *+ β*_*1*_*G*_*1*_ *+ β*_*2*_*A*_*i*_ *+ β*_*3*_*Sex + β*_*4*_*TIV + ε*_*i*_ in this general linear model, we set each regional volume (y_i_) as the objective variable, and the main effect group (G_i_), age (A_i_), gender (Sex), and total intracranial volume (TIV) as covariates.

In addition, we conducted a complementary analysis that compared HD patients who didn’t have comorbid OCD to patients with OCD, to exclude the effects of comorbid OCD condition on HD.

Finally, in order to investigate the relationship between the clinical data (age of onset, symptomatic severity) and the gray matter volume, we conducted a correlation analysis secondarily. As indicators of symptom severity, the SI-R, CIR, and Hoarding dimension score of DY-BOCS were used for the HD patients. Similarly, aggression/checking and contamination/washing scores of DY-BOCS were used as a severity index for the OCD patients. We examined the correlation between each symptom severity index and gray matter volume, and the significance was confirmed by the Spearman’s rank correlation coefficient.

## Results

Details of demographic and clinical data are shown in Table 1. There were no significant differences among HD patients, OCD patients, and HCs in age (HD: 43.9±11.5, OCD: 39.9±9.0, HCs: 42.4±10.4) and gender. Also, there was no difference in the degree of depression and anxiety between the HD and OCD groups. The HD patients showed significantly higher scores on the SI-R and CIR, which means that these HD patients had clinically definite hoarding symptoms. The HD group showed significantly higher hoarding scores on DY-BOCS, while the OCD group showed scores of zero for this dimension. The HD patients had several comorbidities, of which five patients had OCD, three ADHD, and two major depression. For oral administration, there were 10 antidepressants, 7 benzodiazepines, 4 antiepileptics and 2 antipsychotics.

**Table 1:** Demographic and clinical characteristics of all subjects.

	HD(N = 17)	OCD(N = 17)	HCs(N = 17)	p-value
Age	43.9±11.5	39.9±9.0	42.4±10.4	0.53
Male/female	4/13	4/13	4/13	1
Age of onset (range of age)	18.2±12.4 5 − 45	29.8±9.1 15 − 54		<0.01
SI-R	64.3±10.0	−		−
CIR	4.5±1.6	−		−
Y-BOCS	−	24.9±5.6		−
HDRS	3.8±5.3 (N = 16)	4.4±4.2		0.73
HARS	4.9±6.9 (N = 16)	5.5±5.9		0.78
<DY-BOCS>				
Aggression	2.5±3.7	3.1±4.6		0.66
Sexual	1.1±2.7	0.2±0.7		0.18
Symmetry	4.2±3.7	1.8±3.1		0.05
Contamination	4.1±4.1	9.2±4.0		<0.01
Hoarding	7.6±3.6	0		<0.01
Miscellaneous	3.5±4.0	1.4±2.5		0.07
<Comorbidity>				
OCD	5	−		
ADHD	3	0		
ASD	3	0		
Major Depression	2	2		
Anxiety Disorder	4	0		

HD, hoarding disorder; OCD, obsessive-compulsive disorder; HCs, healthy controls; SI-R, Saving Inventory-Revised; CIR, Clutter Image Rating; Y-BOCS, Yale-Brown Obsessive Compulsive Scale; HDRS, Hamilton Depression Rating Scale; HARS, Hamilton Anxiety Rating Scale; DY-BOCS, Dimensional Yale-Brown Obsessive Compulsive Scale; ADHD, attention deficit hyperactive disorder; ASD, autism spectrum disorder

**Fig 1:**
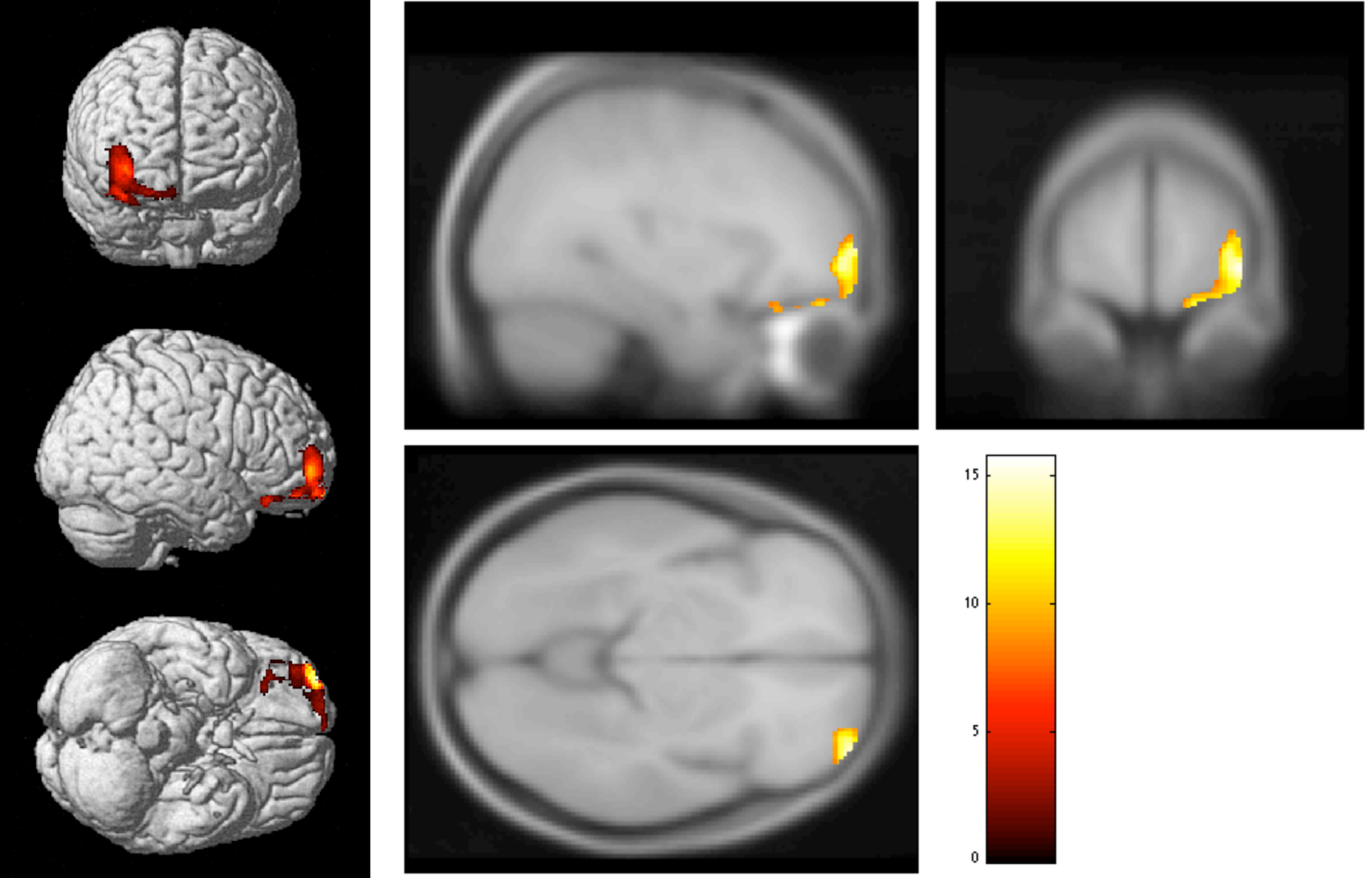
The results of VBM analyses. In voxel-based analysis of variance (ANOVA), three groups (HD, OCD, and HCs) exhibited the presence of significant regional GM volume differences in the right prefrontal regions. The initial voxel threshold was set to 0.001 uncorrected. Clusters were considered as significant when falling below a cluster-corrected p (FWE) = 0.05. The cluster size was 1228 voxels, and the p value was 0.004. Peak coordinates (Montreal Neurological Institute) were x = 20, y = 64, z = -18.

In the results of VBM analyses, all three groups (HD, OCD, and HCs) exhibited the presence of significant regional GM volume differences in the right prefrontal regions, including the frontal pole (FP) and the orbitofrontal cortex (OFC). The initial voxel threshold was set to 0.001 uncorrected. Clusters were considered as significant when falling below a cluster-corrected p (FWE) = 0.05. The cluster size was 1228 voxels and p value was 0.004. Peak coordinates (Montreal Neurological Institute) were x = 20, y = 64, and z = -18 (Fig 1 and Table 2).

**Table 2:** Differences in GM volumes among three groups (HD, OCD, and HCs).

Comparison	BA	Anatomical Region	p value	Cluster size	Peak coordinates (x, y, z)
ANOVA	11	R. superior frontal gyrus	0.004	1228	20 64–18

ANOVA, analysis of variance; BA, Brodmann area; HD, hoarding disorder; OCD, obsessive-compulsive disorder; HCs, healthy controls

**Fig 2:**
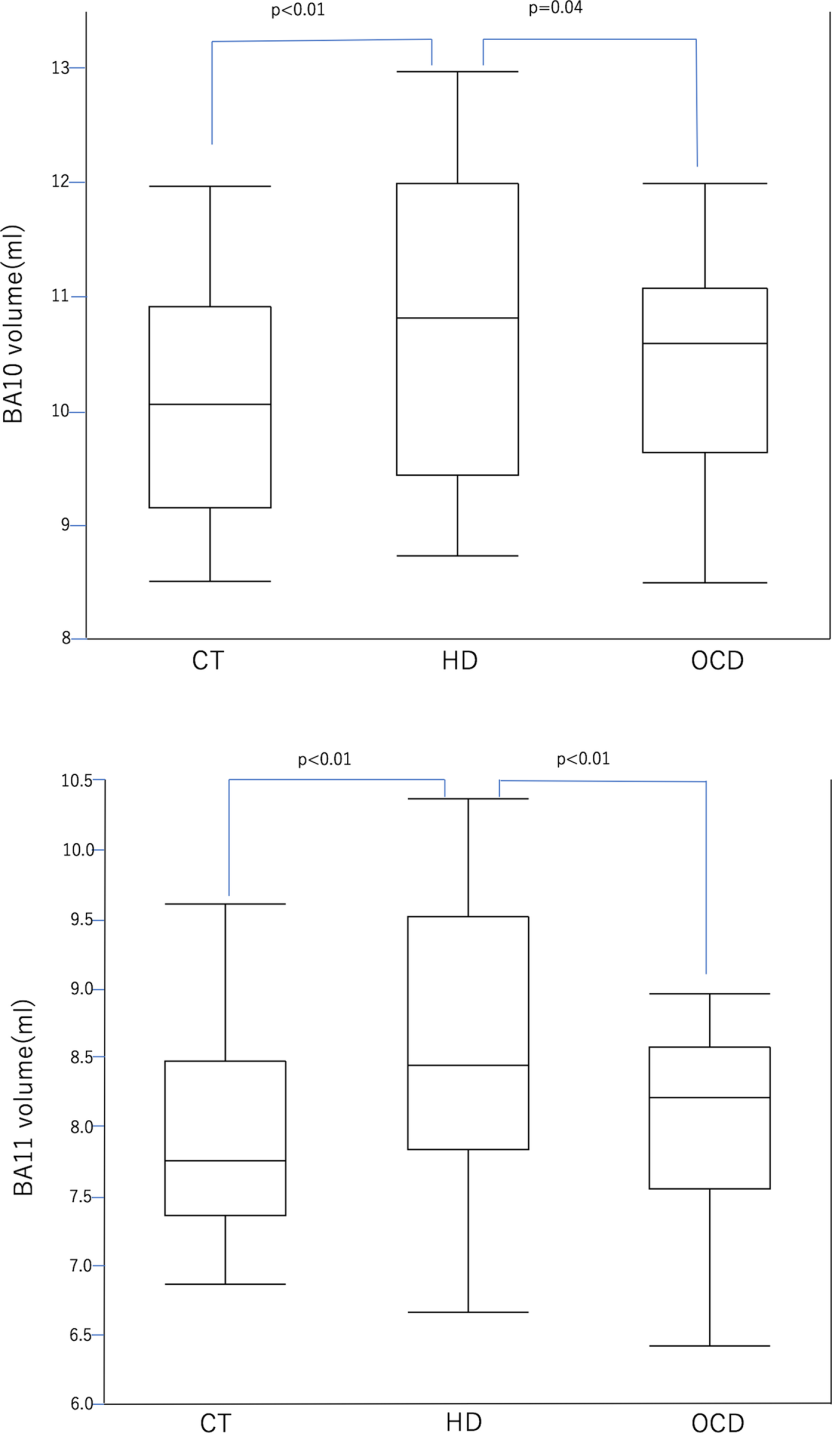
The results of ROI analysis. In the comparison of the three groups based on the gray matter volume obtained by ROI analysis, the HD group showed significantly increased GM volumes compared to the OCD group and the healthy control group (p<0.05) in both BA10 and BA11.

**Fig 3:**
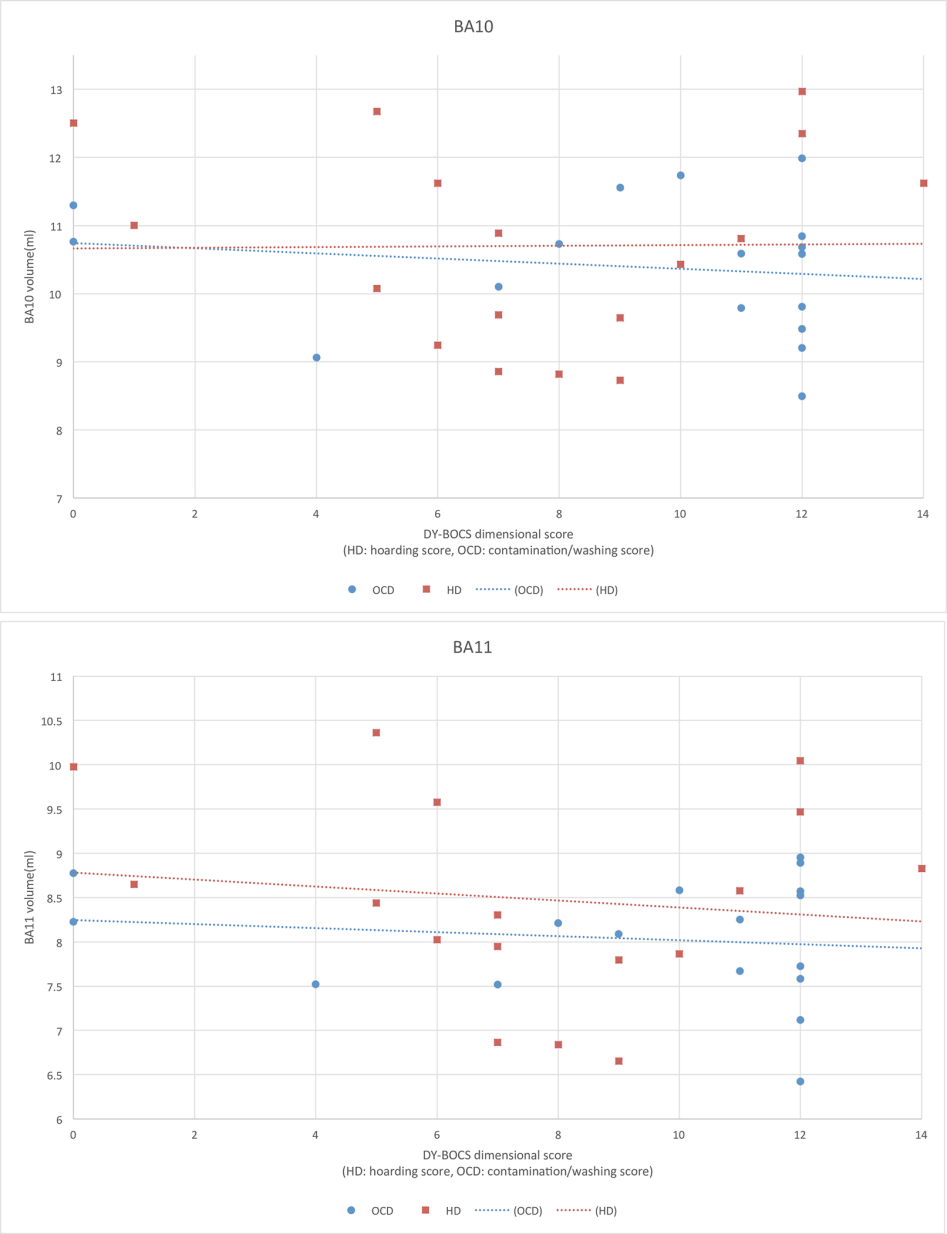
The results of correlation analysis. No significant correlation between the DY-BOCS dimensional score (HD: hoarding score, OCD: contamination/washing score) and GM volume was observed in either the HD or OCD group.

In the comparison of the three groups based on the gray matter volume obtained by ROI analysis, the HD group showed a significantly increased GM volume compared to the OCD group and the healthy control group (p<0.05) in both BA10 and BA11. There was no significant difference between OCD and the healthy control group (Fig 2). In the complementary analysis that compared the HD group without OCD to the OCD group, the volume increase of the HD group in BA10 and BA11 remained significant. A correlation analysis that investigated the relationship between the clinical data and the gray matter volume revealed no significant correlation between the clinical data including age of onset and symptom severity scores, and GM volume in both HD and OCD groups (Fig 3).

## Discussion

In the present study, we found that the HD patients showed an increased GM volume in the broad prefrontal regions including the FP and OFC compared with the OCD patients. On the other hand, a human lesion study[1] showed an association between the prefrontal region and collecting behavior. Actually, the relationship between brain function and volumetric change is still unknown. An et al.　reported that the OCD patients with prominent hoarding symptoms showed greater activation in the bilateral anterior VMPFC (BA11/10) than patients without hoarding symptoms and healthy controls[4]. Although they found excessive activation in these regions, they noted that“dysfunction in these regions (regardless its cause) seems to be associated with abnormal hoarding behaviors”. Similarly, we think that it is still unknown how the increased GM volume in the prefrontal region is associated with dysfunction of the same site and hoarding behaviors. The FP (BA10) comprises the most anterior part of the frontal lobe. The FP is assumed to be related to various brain functions, such as multi-tasking[20], cognitive branching[21], prospective memory[22], conflict resolution[23], and selection of sub-goals[24]. As far as we know, there is no study of HD that found any anatomical abnormalities related to these cognitive dysfunctions. By using functional neuroimaging, Tolin et al. found that patients with hoarding symptoms showed biphasic abnormalities in the insula and anterior cingulate cortex (ACC)[7]. However, they did not find functional abnormalities in the prefrontal regions due to the nature of the task they employed. Meanwhile, by examining the healthy human participants, Fleming et al. reported that GM volume in the right anterior FP involved in the ability of metacognition[25]. Schmitz et al. also suggested that a more accurate level of trait/ability-based insight was related to increased signal change in the right anterior dorsal prefrontal cortex in traumatic brain injury patients[26]. Our speculation is that the function of the right prefrontal cortex might be involved in insight as a kind of metacognition. It would be reasonable that function abnormality in this area also affects decision-making such as acquiring, organizing, and throwing away objects. We, however, need further examination.

OFC has been frequently suggested to be associated with the pathophysiology of OCD. Although one Mega-Analysis study[27] suggested that OCD patients had significantly smaller volumes of frontal gray and white matter bilaterally, the results in VBM studies so far are inconsistent. For example, several studies have indicated that OCD patients have a larger OFC compared to control groups[8] [28] [29], while other studies report a smaller OFC[30] [31] [32]. In the present study, the HD group showed a prominent increase of OFC volume compared to the OCD group. Perhaps this result might help to explain the inconsistency of previous studies for OCD. By using a neurocognitive task during fMRI, Tolin et al. found that OCD patients, but not HD patients, were characterized by excessive activity in the left and right orbitofrontal gyri[33]. Although OCD is recognized as a disease of strong heterogeneity, most previous studies have included various subtypes of OCD and analyzed them as one group. Thus, it is undeniable that a mixture of hoarding symptoms might have influenced the results of these studies. To the extent that we have investigated, past VBM studies included approximately 15% to 55%[8] [30] [32] [34] [35] [36] OCD patients with hoarding symptoms. Since having any hoarding symptoms has been considered a subtype of OCD until primary hoarding symptoms were defined as HD in DSM-5, there is a possibility that the primary and secondary hoarding symptoms of OCD were completely mixed as “OCD”. Therefore, our result showing a significant increase in the prefrontal region in HD compared to OCD without hoarding has important implications.

Like OCD, HD is considered to have cognitive dysfunction as the basis of the disease. In recent years, research findings on various cognitive dysfunctions and neural bases of HD have been accumulated. With regard to cognitive dysfunction, impairments in various areas such as attention, decision-making, categorization and verbal and nonverbal recall have been clarified[37] [38] [39] [40] [41]. This result is convincing after considering the clinical features of HD and suggests that the structural abnormality in the prefrontal regions might be related to the pathophysiology of HD.

Comparison between OCD patients and HCs showed no significant differences. The OCD patients participating in this study were selected from the OCD patients participating in a previous study [42] by our group. Our previous study showed that OCD patients have increased GM volume in left thalamus compared to healthy controls, and this result is consistent with previous findings. Therefore, several reasons, such as sample size and excluding OCD with hording symptoms, might have affected the results.

A correlation between clinical data and GM volume has been mentioned in　previous studies. A mega-analysis study showed that adult OCD patients with an early onset exhibited larger pallidum volumes and patients with a late onset exhibited smaller hippocampal volumes than controls[43]. Likewise, the correlation between age of onset and GM volume was pointed out in our previous study[42]. Alvarenga et al. showed that scores on the “hoarding” dimension of DY-BOCS were positively correlated with GM volume in the left superior lateral OFC and negatively correlated in the right parahippocampal gyrus, while there was no correlation between contamination dimension and GM volume[10]. Meanwhile, no correlation between the clinical data, including age of onset and symptom severity scores and GM volume, was found in our study. This might be affected by differences such as sample size and comorbidity, as described later.

The criteria of hoarding disorder (HD) in the previous studies employed various diagnostic criteria such as the clinical criteria outlined by Frost and Hartl in1996 and proposed for DSM-5[7], lifetime hoarding symptoms on the Y-BOCS Symptom Checklist and scored above the group median on the SI-R (SI-R>30) [4] and HD criteria in DSM-5[11]. Although they have not been unified, most of the previous studies recruited homogeneous subjects with hoarding and shared common recognition that HD has different pathology from typical OCD. Therefore, we thought that comparing our results with previous studies has a certain meaning to comprehend the pathology of HD.

Our study had several limitations. The hoarding sample was relatively small and had various comorbidities such as OCD, ADHD, and major depression. These comorbid disorders are suggested to cause specific volumetric changes. It is suggested that patients with major depression disorder have smaller volumes of specific regions, such as the basal ganglia, thalamus, hippocampus, frontal lobe, orbitofrontal cortex, and gyrus rectus [44], and it is known that ADHD also shows a volume reduction in the right lentiform nucleus that is extended to the caudate nucleus [45]. We did not especially exclude patients with concurrent OCD. There were five patients with concurrent HD and OCD in this study, and it is undeniable that this might have certain effects on the results. Concerned with this limitation, we conducted a second analysis with pure HD patients without OCD and obtained results similar to the main analysis, suggesting that they had an increased volume of the prefrontal region compared to OCD without hoarding. A further increase in the sample size and analysis of unalloyed HD patients who are not complicated by OCD are needed in the future. Finally, we were not able to control the effects of drug therapy in this study. Many patients took medications such as antidepressants and benzodiazepines, so the effects might need to be considered.

## Supporting information

S1 Appendix The detailed information of HD patients. (XLSX)

S2 Appendix The detailed information of OCD patients. (XLSX)

S3 Appendix The detailed information of healthy controls. (XLSX)

S1 Fig The result of ANOVA with SPM software. (PNG)
