# Supplementary figures and images for: A unique increase in prefrontal gray matter volume in hoarding disorder compared to obsessive-compulsive disorder
Source: PLoS One. 2018 Jul 16;13(7):e0200814. doi: 10.1371/journal.pone.0200814 (PMC6047811; doi:10.1371/journal.pone.0200814)

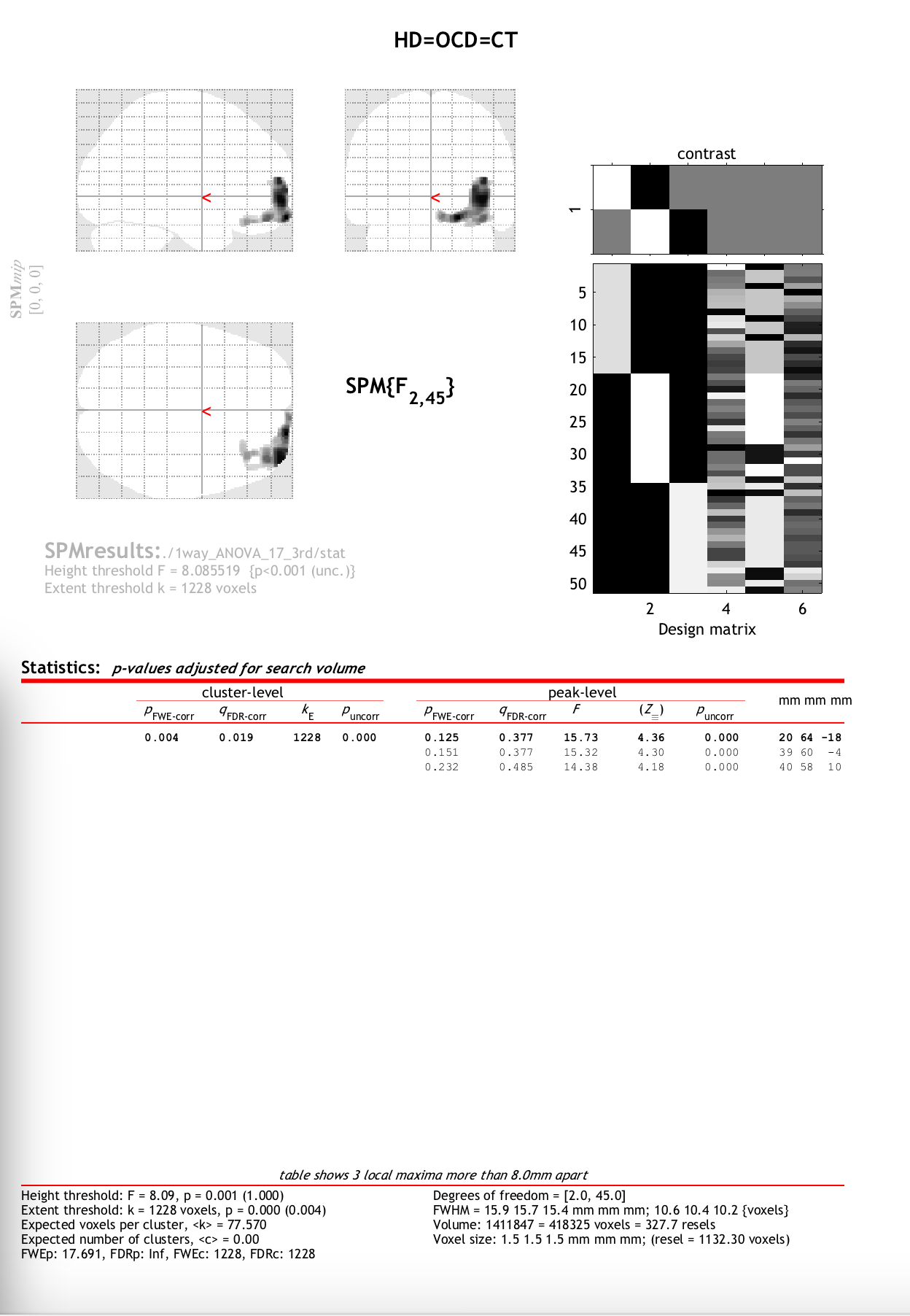

Supplement: S1 Fig — (PNG) [file pone.0200814.s004.png]
